# Anti-Hangover and Hepatoprotective Effects of the Leaf Extract of *Thunbergia laurifolia* in Sprague–Dawley Rats

**DOI:** 10.3390/ph18050685

**Published:** 2025-05-05

**Authors:** Supaporn Intatham, Weerakit Taychaworaditsakul, Phraepakaporn Kunnaja, Ariyaphong Wongnoppavich, Kanjana Jaijoy, Sunee Chansakaow, Piyanuch Rojsanga, Seewaboon Sireeratawong

**Affiliations:** 1 Clinical Research Center for Food and Herbal Product Trials and Development (CR-FAH), Faculty of Medicine, Chiang Mai University, Chiang Mai 50200, Thailand; intatham_s@outlook.com; 2 Department of Pharmacology, Faculty of Medicine, Chiang Mai University, Chiang Mai 50200, Thailand; 3 Department of Biochemistry, Faculty of Medicine, Chiang Mai University, Chiang Mai 50200, Thailand; weerakit.tay@cmu.ac.th (W.T.); ariyaphong.w@cmu.ac.th (A.W.); 4 Department of Medical Technology, Faculty of Associated Medical Sciences, Chiang Mai University, Chiang Mai 50200, Thailand; phraepakaporn.k@cmu.ac.th; 5 McCormick Faculty of Nursing, Payap University, Chiang Mai 50000, Thailand; joi.kanjana@gmail.com; 6 Department of Pharmaceutical Sciences, Faculty of Pharmacy, Chiang Mai University, Chiang Mai 50200, Thailand; sunee.c@cmu.ac.th; 7 Department of Pharmaceutical Chemistry, Faculty of Pharmacy, Mahidol University, Bangkok 10400, Thailand; piyanuch.roj@mahidol.ac.th

**Keywords:** *Thunbergia laurifolia*, hangover, alcohol metabolism, ethanol, acetate, alcohol dehydrogenase, aldehyde dehydrogenase, antioxidant, hepatoprotective

## Abstract

**Background/Objectives:** The present study aims to evaluate the anti-hangover and hepatoprotective activities of the leaf extract of *T. laurifolia* in experimental animals. **Methods:** Two experiments were conducted that involved giving a single dose of the leaf extract of *T. laurifolia* (1, 10, or 100 mg/kg body weight) to rats 30 min either before or after administration of 40% ethanol (5 g/kg body weight). The locomotor activity of the rats was measured before and after receiving the test substances. Blood samples were collected to determine the ethanol, acetate, and liver enzyme levels. Liver tissues were collected to evaluate alcohol-metabolizing enzymes, antioxidant enzyme activities, and antioxidant levels. **Results:** Administration of the leaf extract of *T. laurifolia* to the rats prior to ethanol increased locomotor activity and reduced blood ethanol levels. The extract also prevented changes in liver enzyme levels and demonstrated antioxidant activity by scavenging free radicals resulting from ethanol-induced oxidative stress. Conversely, rats administered the leaf extract of *T. laurifolia* after receiving ethanol were able to reduce the elevated liver enzyme levels back to normal levels, and probably helped to inhibit the harmful effects of free radicals by stimulating the synthesis and/or activities of antioxidant enzymes. Administration of the leaf extract of *T. laurifolia* either before or after ethanol exposure was able to reduce the activity of an alcohol-metabolizing enzyme as well as reduce blood acetate levels. **Conclusions:** In summary, receiving the leaf extract of *T. laurifolia* before alcohol consumption could probably help to reduce hangover symptoms and was shown to have hepatoprotective effects superior to receiving the extract after alcohol consumption.

## 1. Introduction

Consumption of alcoholic beverages, especially those containing ethanol, can have adverse health effects. Alcohol is a neurotoxin that can cause both neurodegeneration and neuroinflammation [1]. Excessive alcohol consumption can cause negative short-term side effects, such as a hangover, while chronically drinking alcohol can cause negative long-term side effects, such as alcohol addiction and alcoholic liver disease [2,3,4]. Additionally, consuming alcoholic beverages can cause lifestyle diseases such as cancer [5]. According to the World Health Organization (WHO), total alcohol per capita consumption (APC) worldwide declined between 2010 and 2019. Nevertheless, subregional comparisons show that Southeast Asia and Southern Europe have seen an increase in their alcohol consumption over that period. This increase is likely to have resulted from economic growth, as well as from people’s refusal to comply with alcohol control policies [6]. Furthermore, in 2019, it was found that 2.6 million people worldwide died as a result of alcohol consumption, and more than 2 million of these were men. Appallingly, young adults (20–39 years old) accounted for the largest proportion of alcohol-attributable deaths, at 13%. [7].

One of the common short-term effects of excessive alcohol consumption is a hangover [2]. An alcohol hangover, as defined by the Alcohol Hangover Research Group in 2016 [8], is a combination of negative symptoms, both psychological and physical, that result from a single large amount of alcohol consumption, which typically begins when an initially high blood alcohol concentration (BAC) declines to near zero. Physical hangover symptoms that appear in heavy drinkers include fatigue, headache, sensitivity to light and sound, muscle aches, and thirst [9]. In addition, mental or psychological symptoms associated with hangovers can also include mood swings, anxiety, and depression [10]. More than 90% of alcohol elimination from the human body relies on oxidation in the liver, and two liver enzymes play a crucial role in this process. First, alcohol dehydrogenase (ADH) oxidizes ethanol to acetaldehyde. Then, aldehyde dehydrogenase (ALDH) oxidizes acetaldehyde to acetate [11]. It was noteworthy that a previous study in an animal model reported that acetate is involved in causing hangover headaches [12]. Ethanol has also been reported to influence locomotor activity in animals [13]. Prior studies in animals and humans have found that alcohol intake causes changes in liver enzyme levels, including aspartate aminotransferase (AST), alanine aminotransferase (ALT), and alkaline phosphatase (ALP), which are markers of liver function [14,15]. Alcohol consumption also triggers the production of free radicals, which causes oxidative stress, leading to inflammation and damage to the liver, which, in turn, contributes to various diseases such as alcohol-associated cirrhosis as well as liver cancer [16].

*T. laurifolia* (Family Acanthaceae) is a medicinal plant that grows widely in tropical and mixed forests in Thailand [17]. This plant is known as Rang Chuet in Thai, and it has been used as an antipyretic in traditional Thai medicine [17,18]. Both stems and roots of *T. laurifolia* have been traditionally utilized to make herbal teas to help detoxify from alcohol and food poisoning [19,20]. Various pharmacological activities of *T. laurifolia* have been reported, such as wound healing [21], antitumor [22], antifibrotic [23], antidiabetic [24], and antioxidant activities [20,25,26]. Furthermore, post-treatment with the aqueous extract of *T. laurifolia* leaves has been shown to normalize AST and ALT enzyme levels, promoting hepatic cell recovery from alcohol-induced liver injury in rats [27]. Recent toxicity studies have found no mortality and no significant changes in animal behaviors, gross pathology, and histopathology in both an acute toxicity study in which the rats received aqueous, ethanol, or acetone extracts of *T. laurifolia* leaves in a dose of 15,000 mg/kg body weight, and in a sub-chronic toxicity study of rats treated with aqueous or ethanol extracts of *T. laurifolia* leaves at doses of up to 5000 mg/kg body weight for 90 days [18].

Obviously, these findings strongly suggest that the leaf extract of *T. laurifolia* appears to be relatively safe and that it has an ameliorative effect against alcohol-induced liver damage. The present study aimed to investigate the anti-hangover and hepatoprotective activities of the leaf extract of *T. laurifolia* to assess the potential for its development as an anti-hangover product. It is hypothesized that *T. laurifolia* may help prevent liver damage due to its antioxidant effects.

## 2. Results

### 2.1. Chemical Profile and Quantitative Analysis of the Dried Leaves and the Leaf Extract of T. laurifolia

**Figure 1:**
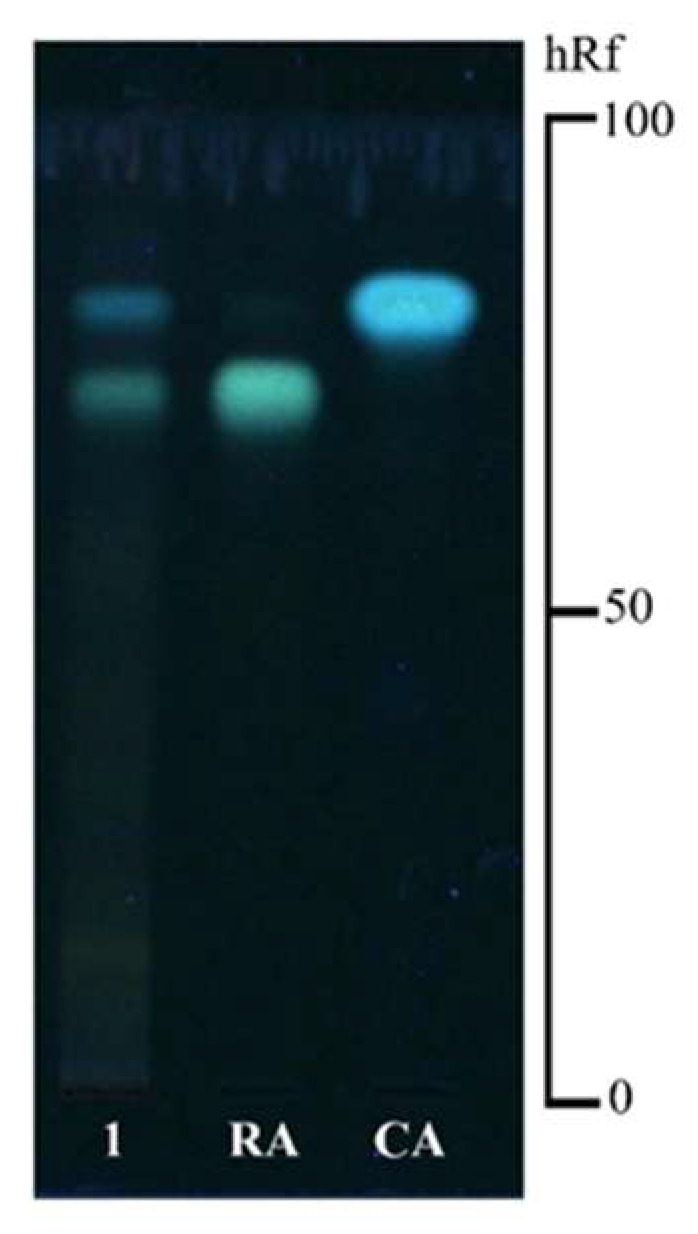
TLC chromatogram of the dried leaves of *T. laurifolia* after being sprayed with NP/PEG reagent and visualized under UV light at 366 nm; 1 = the dried leaves of *T. laurifolia*, RA = rosmarinic acid and CA = caffeic acid.

The chemical profile of the dried leaves of *T. laurifolia* was identified by thin-layer chromatography (TLC) with detection under UV light at 366 nm after spraying with a natural product/polyethylene glycol (NP/PEG) reagent. The two chemical reference standards, caffeic acid and rosmarinic acid, were represented as blue fluorescent spots at hRf values of 85 and 71, respectively. TLC analysis (Figure 1) confirmed the presence of caffeic acid (hRf 85) and rosmarinic acid (hRf 71) in the *T. laurifolia* leaf extract by comparison with reference standards. Additionally, the quantitative analysis of marker contents by high-performance liquid chromatography (HPLC) revealed the presence of caffeic acid and rosmarinic acid at 0.12% and 0.30% (*w*/*w*), respectively, in the dried leaves of *T. laurifolia* (Table 1).

**Figure 2:**
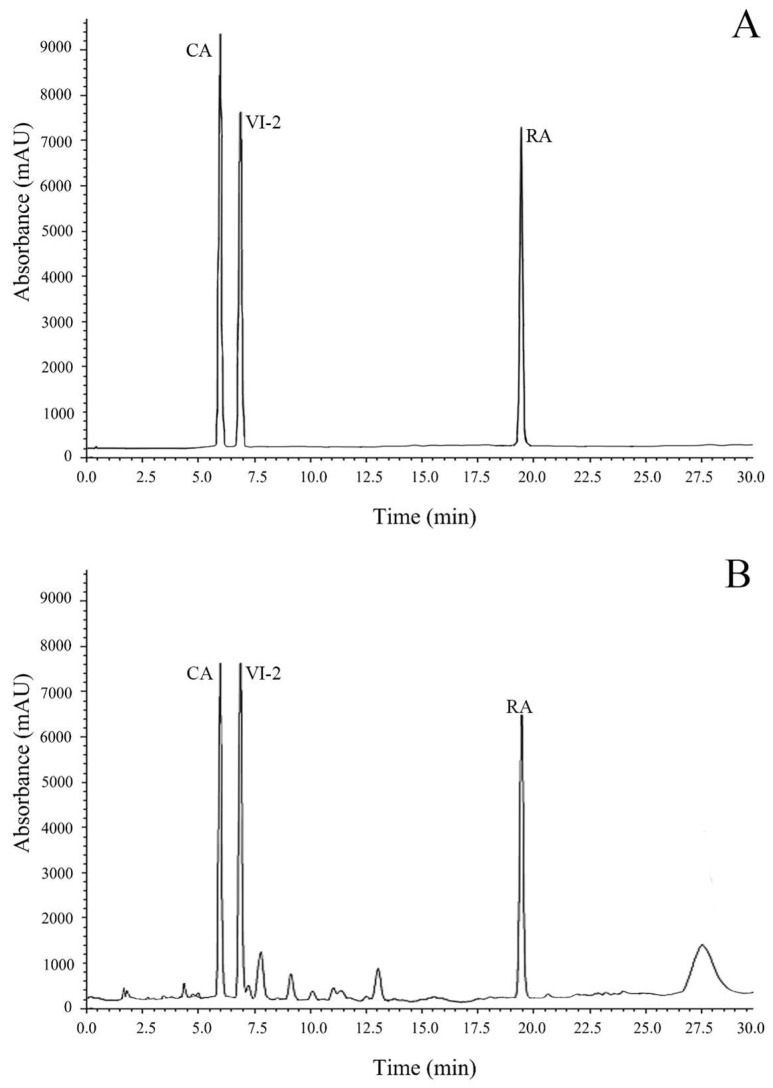
HPLC chromatograms of standard solution (**A**) and the leaf extract of *T. laurifolia* (**B**). CA = caffeic acid, RA = rosmarinic acid, and VI-2 = vicenin-2.

Figure 2 demonstrates the HPLC chromatogram of the leaf extract of *T. laurifolia* containing caffeic acid (retention time (RT) = 6.0 min, resolution = 2.3) and rosmarinic acid (RT = 19.5 min, resolution = 3.0). Caffeic acid and rosmarinic acid contents were measured as 0.14% (*w*/*w*) and 0.24% (*w*/*w*), respectively. In this study, only the contents of caffeic acid and rosmarinic acid were examined as the analytical markers to control the consistency of the extract. Nevertheless, vicenin-2, detected at a retention time of 6.8 min, was found after the study was completed by comparing the UV spectrum and retention time to those of the standard solution. Table 1 illustrates that the leaf extract of *T. laurifolia*, obtained after spray-drying, had a dark brown color and a distinct odor. The weight loss of the leaf extract of *T. laurifolia* at 105 °C was 5.98%. These characteristics could be employed to develop the specifications for standardized *T. laurifolia* leaf extract. The specifications would ensure consistency in the quality of the extract for further use.

### 2.2. Motor Activity of Rats Administered the Leaf Extract of T. laurifolia Before or After Receiving Ethanol

**Figure 3:**
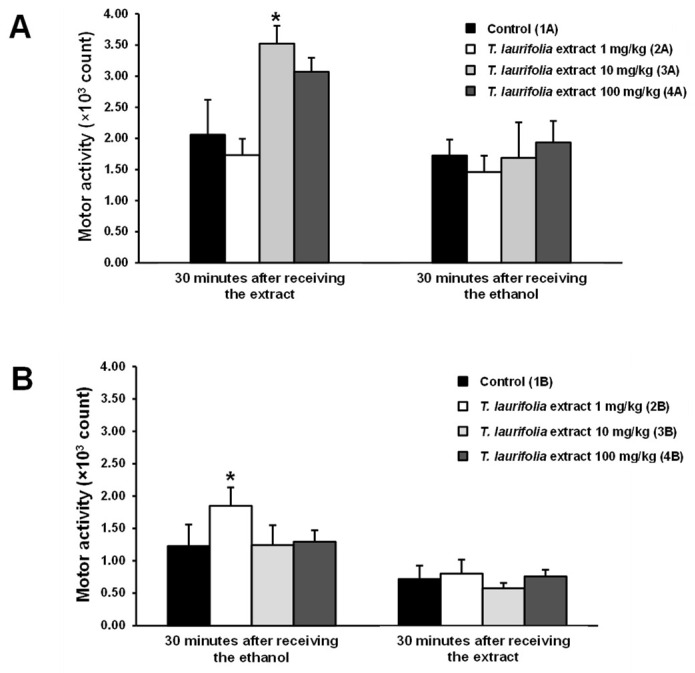
Motor activity of rats administered the leaf extract of *T. laurifolia* before receiving ethanol (**A**) and rats administered ethanol before receiving the leaf extract of *T. laurifolia* (**B**). The data are expressed as mean ± S.E.M. (*n* = 6 rats per group). * *p* < 0.05 represents statistically significant differences from the control group.

No statistically significant differences in motor activity of all rats in either the pre-treatment or the post-treatment experiment were found before the start of the experiment. In the pre-treatment experiment, group 2A, receiving 1 mg/kg of the leaf extract of *T. laurifolia,* had decreased motor activity 30 min after extract administration, but this was not a statistically significant difference compared to the control group. Whereas the rats in groups 3A and 4A that received 10 and 100 mg/kg of the leaf extract of *T. laurifolia*, respectively, were found to have increased motor activity 30 min after the extract administration, with the 3A group having a statistically significant difference compared to the control group. This suggests that the middle dose of the extract (10 mg/kg extract) may have a stimulating effect on motor activity. In addition, 30 min after feeding ethanol to the rats, the rats in groups 2A (1 mg/kg extract) and 3A (10 mg/kg extract) had decreased motor activity, whereas group 4A (100 mg/kg extract) showed increased motor activity but were not statistically significantly different compared to the control group (Figure 3A). In the post-treatment experiment, 30 min after receiving ethanol, all groups of rats had motor activity that was not different from the control group, except for group 2B (1 mg/kg extract), which had a statistically significant increase in motor activity. Subsequently, 30 min after receiving the leaf extract of *T. laurifolia*, the rats in groups 2B, 3B, and 4B that received 1, 10, and 100 mg/kg of the leaf extract of *T. laurifolia*, respectively, showed an obvious reduction in motor activity compared to motor activity measured 30 min after ethanol administration. Nonetheless, no statistically significant difference in motor activity was found between the groups after 30 min of the extract administration (Figure 3B).

### 2.3. Blood Ethanol Levels of Rats Administered the Leaf Extract of T. laurifolia Before or After Ethanol Administration

There was no statistically significant difference in the blood ethanol levels in either the pre-treatment or the post-treatment experiment at 0.5 h. In the pre-treatment experiment, at the first hour, the rats in groups 2A (1 mg/kg extract) and 3A (10 mg/kg extract) had decreased blood ethanol levels but had no statistically significant difference when compared to the control group. However, group 4A (100 mg/kg extract) had statistically significantly reduced blood ethanol levels when compared to the control group. At the third hour, blood ethanol levels were found to be increased in all groups of rats compared to the first hour. Then, at the fifth hour, all groups of rats had decreased blood ethanol levels compared to the third hour but were not statistically significantly different from the control group (Table 2). In the post-treatment experiment, at the first hour, the blood ethanol levels of the rats treated with the leaf extract of *T. laurifolia* after ethanol were not different from those of the control group, except for group 3B, which received 10 mg/kg of the extract, which had a statistically significant decrease in blood ethanol levels compared to the control group. After that, all groups of rats were found to have increased blood ethanol levels at the third hour compared to the first hour. At the fifth hour, the blood ethanol levels of all groups of rats were decreased compared to the third hour. Furthermore, a statistically significant decrease in the blood ethanol levels was observed in group 3B (10 mg/kg extract) compared to the control group at both the third and fifth hours (Table 3).

### 2.4. ADH Activity in Liver Tissues from Rats Administered the Leaf Extract of T. laurifolia Before or After Receiving Ethanol

**Figure 4:**
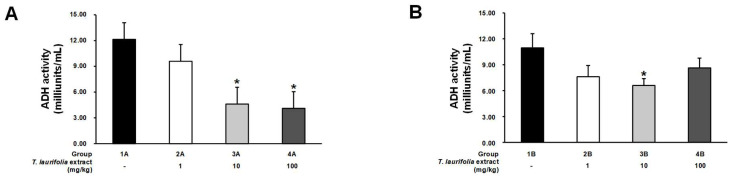
Alcohol dehydrogenase (ADH) activity in liver tissues from rats administered the leaf extract of *T. laurifolia* before receiving ethanol (**A**) and rats administered ethanol before receiving the leaf extract of *T. laurifolia* (**B**). The data are expressed as mean ± S.E.M. (*n* = 6 rats per group). * *p* < 0.05 represents statistically significant differences from the control group.

In the pre-treatment experiment, the leaf extract of *T. laurifolia* dose-dependently decreased ADH activity, with groups 3A and 4A receiving 10 and 100 mg/kg extract, respectively, having a statistically significant decrease in ADH activity compared to the control group (Figure 4A). In a post-treatment experiment, the rats receiving all doses of the leaf extract of *T. laurifolia* had decreased ADH activity, with a statistically significant decrease in group 3B receiving 10 mg/kg extract compared to the control group (Figure 4B).

### 2.5. ALDH Activity in Liver Tissues from Rats Administered the Leaf Extract of T. laurifolia Before or After Receiving Ethanol

**Figure 5:**
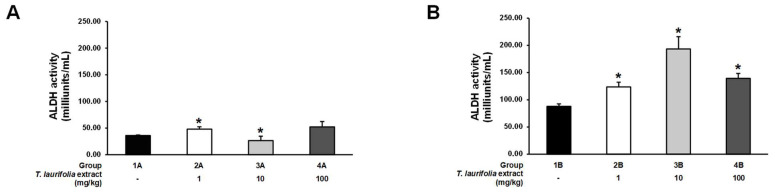
Aldehyde dehydrogenase (ALDH) activity in liver tissues from rats administered the leaf extract of *T. laurifolia* before receiving ethanol (**A**) and rats administered ethanol before receiving the leaf extract of *T. laurifolia* (**B**). The data are expressed as mean ± S.E.M. (*n* = 6 rats *per* group). * *p* < 0.05 represents statistically significant differences from the control group.

In the pre-treatment experiment, an increase in ALDH activity was observed in groups 2A and 4A receiving the leaf extract of *T. laurifolia* at doses of 1 and 100 mg/kg, respectively, before ethanol exposure, with a statistically significant difference in ALDH activity found in group 2A compared to the control group. Meanwhile, group 3A was found to have statistically significantly reduced ALDH activity when receiving 10 mg/kg of the leaf extract of *T. laurifolia* prior to ethanol (Figure 5A). In the post-treatment experiment, it was found that all doses of the leaf extract of *T. laurifolia* administered to rats after ethanol exposure statistically significantly increased the ALDH activity compared to the control group (Figure 5B).

### 2.6. Blood Acetate Levels of Rats Administered the Leaf Extract of T. laurifolia Before or After Ethanol Administration

No statistically significant difference between groups in blood acetate levels was observed at 0.5 h in either the pre-treatment or the post-treatment experiment. In the pre-treatment experiment, a statistically significant decrease in blood acetate levels at the first hour was observed in groups 3A and 4A, which received 10 and 100 mg/kg of the extract, respectively. At the third hour, the rats administered all doses of the extract before ethanol had statistically significantly reduced blood acetate levels compared to the control group (Table 4). In the post-treatment experiment, at the first, second, and third hours, it was found that the rats in group 3B receiving 10 mg/kg of the extract after ethanol exposure had a statistically significant decrease in blood acetate levels compared to the control group (Table 5).

### 2.7. Liver Enzyme Levels of Rats Administered the Leaf Extract of T. laurifolia Before or After Ethanol Administration

In the pre-treatment experiment, no statistically significant differences in AST, ALT, as well as ALP levels were found in the rats that received different doses of the leaf extract of *T. laurifolia* before receiving ethanol compared to the control group (Table 6). In the post-treatment experiment, the rats in groups 2B and 3B that received ethanol before the leaf extract of *T. laurifolia* at doses of 1 and 10 mg/kg, respectively, had a statistically significant reduction in their AST levels compared to the control group. Whereas statistically significantly lower levels of AST and ALT were observed in the 4B group administered ethanol before receiving 100 mg/kg of the leaf extract of *T. laurifolia* (Table 7).

### 2.8. Superoxide Dismutase (SOD) Activity in Liver Tissues from Rats Administered the Leaf Extract of T. laurifolia Before or After Receiving Ethanol

**Figure 6:**
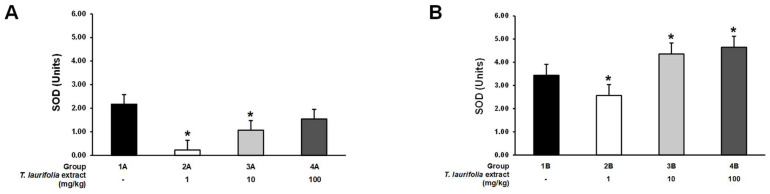
Superoxide dismutase (SOD) activity in liver tissues from rats administered the leaf extract of *T. laurifolia* before receiving ethanol (**A**) and rats administered ethanol before receiving the leaf extract of *T. laurifolia* (**B**). The data are expressed as mean ± S.E.M. (*n* = 6 rats per group). * *p* < 0.05 represents statistically significant differences from the control group.

In the pre-treatment experiment, groups 2A and 3A of the rats that were administered the extract at doses of 1 and 10 mg/kg, respectively, had a statistically significant decrease in SOD activity compared to the control group (Figure 6A). In the post-treatment experiment, a statistically significant decrease in SOD activity was observed in the 2B group (1 mg/kg extract). On the other hand, the rats in groups 3B (10 mg/kg extract) and 4B (100 mg/kg extract) had a statistically significant increase in the activity of SOD when compared to the control group (Figure 6B).

### 2.9. Catalase (CAT) Activity in Liver Tissues from Rats Administered the Leaf Extract of T. laurifolia Before or After Receiving Ethanol

**Figure 7:**
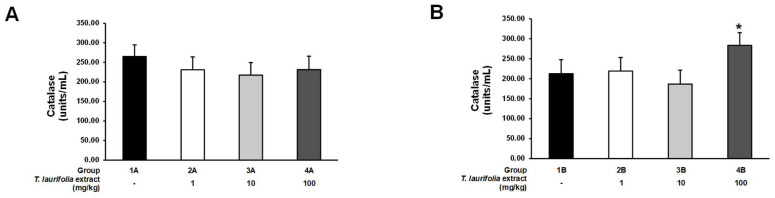
Catalase (CAT) activity in liver tissues from rats administered the leaf extract of *T. laurifolia* before receiving ethanol (**A**) and rats administered ethanol before receiving the leaf extract of *T. laurifolia* (**B**). The data are expressed as mean ± S.E.M. (*n* = 6 rats per group). * *p* < 0.05 represents statistically significant differences from the control group.

In the pre-treatment experiment, a decrease in CAT activity was found in the rats receiving all doses of the leaf extract of *T. laurifolia* before ethanol compared to the control group; however, these differences were not statistically significant (Figure 7A). In the post-treatment experiment, there was no statistically significant difference in the activity of CAT in groups 2B and 3B receiving ethanol prior to the leaf extract of *T. laurifolia* at doses of 1 and 10 mg/kg, respectively, when compared to the control group. Nonetheless, a statistically significant increase in CAT activity was observed in the 4B group that received ethanol before 100 mg/kg of the extract (Figure 7B).

### 2.10. Total Glutathione Levels in Liver Tissues from Rats Administered the Leaf Extract of T. laurifolia Before or After Receiving Ethanol

**Figure 8:**
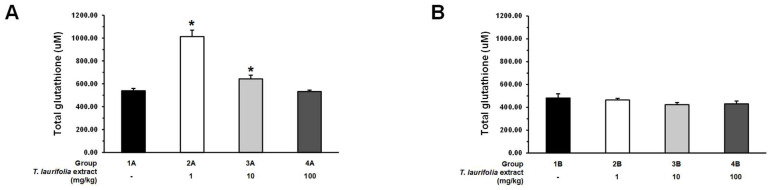
Total glutathione levels in liver tissues from rats administered the leaf extract of *T. laurifolia* before receiving ethanol (**A**) and rats administered ethanol before receiving the leaf extract of *T. laurifolia* (**B**). The data are expressed as mean ± S.E.M. (*n* = 6 rats per group). * *p* < 0.05 represents statistically significant differences from the control group.

In the pre-treatment experiment, groups 2A and 3A that received 1 and 10 mg/kg of the leaf extract of *T. laurifolia*, respectively, before ethanol exposure, had a statistically significant increase in the levels of total glutathione. Whereas the rats in group 4A were fed the extract at a dose of 100 mg/kg before ethanol had lower total glutathione levels than groups 2A and 3A; however, there was no statistically significant difference when compared to the control group (Figure 8A). In the post-treatment experiment, the administration of all doses of the leaf extract of *T. laurifolia* to rats after ethanol exposure decreased the total glutathione levels but did not show any statistically significant difference when compared to the control group (Figure 8B).

## 3. Discussion

For a long time, alcohol has been used in the preparation of different types of beverages, which have more harmful effects than benefits in the long-term effects on health [6]. Alcohol consumption is a leading cause of death, traffic injuries, increased risk of various diseases and illnesses, domestic violence, and child maltreatment [28,29,30]. When alcohol enters the body through ingestion, it undergoes various processes, starting with absorption in the digestive system. After that, alcohol is distributed to other organs. Then, it is metabolized by alcohol-metabolizing enzymes and is then excreted from the body in both its original form and its metabolites [31,32]. The effects of alcohol on the nervous system can include intoxication and can result in personality and behavior changes [33]. The severity of these symptoms varies depending on each person’s physical health condition [34]. Therefore, focusing research to find the potential substances to relieve hangover symptoms and counteract alcohol toxicity is considered an important challenge.

According to Thai traditional medicine wisdom, *T. laurifolia*, known as Rang Chuet in Thai, has long been used to make herbal teas and drunk to detoxify from alcohol and food poisoning [19,20]. Currently, many studies have reported various pharmacological properties of *T. laurifolia* [20,21,22,23,24,25,26]. One recent study reported that aqueous and ethanolic extracts of *T. laurifolia* leaves at a concentration of 5000 mg/kg did not cause abnormalities or mortality in experimental animals in a sub-chronic toxicity study [18]. The present study aimed to study the anti-hangover and hepatoprotective activities of the leaf extract of *T. laurifolia* in an animal model to assess whether it has the potential to be developed into an anti-hangover product.

Each medicinal plant contains various chemical constituents [35]. Thus, the analysis of chemical markers of medicinal plants is considered a crucial step in developing quality control and the standardization of raw materials and plant extracts during the production process [36]. The present study examined the chemical composition of the dried leaves and the leaf extract of *T. laurifolia* using TLC and HPLC methods. The results showed that both the dried leaves and the leaf extract of *T. laurifolia* contained caffeic acid and rosmarinic acid. The results from the present study are consistent with prior studies that reported the presence of caffeic acid, rosmarinic acid, and vicenin-2 in the aqueous extract of *T. laurifolia* leaves [25,37,38].

The brain, a part of the central nervous system, is an organ with a high water content and a rich blood supply. This allows alcohol to spread into the brain, especially around the cortex, so the central nervous system is most affected by alcohol [39]. Once alcohol enters the body, it is absorbed and spreads to the brain within 5 min, and 10 min after that, alcohol begins to affect the brain [40]. People who drink alcohol at concentrations of up to 11 mM may feel relaxed and euphoric because alcohol selectively depresses functions of certain parts of the brain, particularly the reticular activating system, which is responsible for controlling judgment, reflection, restraint, and thought processes [41,42,43]. In the pre-treatment experiment, motor activity in the rats was increased 30 min after receiving the leaf extract of *T. laurifolia*. Following that, an increasing trend in motor activity was again found 30 min after the rats received ethanol. This might be due to prior administration of the extract, probably affecting neurotransmitter receptors in the brain and causing structural changes, resulting in a partially decreased binding of ethanol to the receptors, thus reducing the brain depressant effect of ethanol. In contrast, a previous study demonstrated that the methanol extract of *T. laurifolia* leaves could stimulate the neuronal activity of specific brain areas involved in controlling locomotor behavior in rats [44]. In the post-treatment experiment, all groups of rats had relatively low motor activity 30 min after ethanol administration, consistent with previous research that found dose-dependent depressant effects of ethanol on the rats’ motor activity [45]. However, only the rats in group 2B showed a significant increase in motor activity compared to the other groups 30 min after receiving ethanol, which might be due to the fact that all addictive substances, one of which is ethanol, can stimulate motor activity according to the psychomotor stimulant theory of addiction [46]. In the present study, 30 min after receiving the leaf extract of *T. laurifolia*, the motor activity of all groups of rats was further reduced compared to 30 min after ethanol administration, and the extract-treated group showed no increase in motor activity compared to the control group. This indicated that the leaf extract of *T. laurifolia* could not counteract the depressant effect of ethanol on the brain. That result might be because when ethanol was first administered to the rats, it was able to bind directly to inhibitory neurotransmitter receptors, such as gamma–aminobutyric acid (GABA), and thus produced depressant effects, whereas the extract administered later could not interfere with the binding of ethanol to the receptors.

The liver removes about 90 percent of the alcohol consumed, with the remaining 10 percent being excreted through breath, urine, and sweat [47]. The main process of alcohol metabolism in the liver relies on the enzymes ADH and ALDH, localized in the cytosol and mitochondrial matrix, respectively [48]. Differences in the functioning of ADH in each person result in different alcohol metabolism abilities, which is one of the reasons why the onset of intoxication varies from person to person [49]. The process of alcohol metabolism in the liver involves two steps. First, ethanol is oxidized by ADH to acetaldehyde. Then, acetaldehyde is oxidized by ALDH to acetate, and acetate itself has been reported to be involved in the hangover headache [11,12]. In the pre-treatment experiment, the leaf extract of *T. laurifolia* at low (1 mg/kg) and middle (10 mg/kg) doses decreased blood ethanol levels but was not statistically different compared to the control group, while the high dose of the extract (100 mg/kg) was demonstrated to statistically significantly reduce blood ethanol levels at the first hour in the rats that received the extract prior to ethanol exposure. This was consistent with the motor activity measurements at 1 h, which showed a trend towards increased motor activity in rats receiving the extract before ethanol. This result might be due to pre-treatment, with low and middle doses of the extract having insufficient potency to reduce the gastrointestinal absorption of subsequently administered ethanol or stimulate neuronal activity in the brain [46]. In the post-treatment experiment, from the first to the fifth hours, only the rats that were administered the extract of *T. laurifolia* at the middle dose (10 mg/kg extract) after ethanol intake showed significantly decreased blood ethanol levels compared to the control group. These results indicated that the subsequent administration of low (1 mg/kg extract) and high (100 mg/kg extract) doses of the extract could not reduce blood ethanol levels, which was consistent with the measurements of motor activity at 1 h that observed a decrease in motor activity in the rats that received the extract after ethanol administration. In both experiments, the blood ethanol levels of rats increased at the third hour, probably because of the saturation of ethanol metabolism through enzymatic pathways in the liver, as zero-order kinetics. This indicates that the ethanol metabolism rate remains constant regardless of the blood ethanol level because ADH, which is the enzyme involved in ethanol metabolism, has reached its capacity, resulting in a large amount of ethanol being continuously absorbed into the bloodstream but not being completely metabolized [50]. This is because ethanol is eliminated by zero-order kinetics, in which a constant amount of ethanol is metabolized per unit of time. Therefore, there is no elimination half-life of ethanol [51]. However, in the present study, in which the rats received a high dose of ethanol, a decrease in blood ethanol levels was observed at the fifth hour in both experiments. In both experiments, it was found that the leaf extract of *T. laurifolia* could reduce the activity of ADH, with a more pronounced reduction observed in the pre-treatment experiment. In general, the decrease in enzyme activity can be caused by several factors, such as changes in pH and temperatures and the presence of any inhibitors, which, in the present experiments, were considered to be due to the extract acting as an inhibitor that could bind to the ADH, thus inactivating or reducing its activities [52,53,54]. The previous study of ethanol-induced liver damage reported that as ADH activity decreases, ALDH activity also decreases in the same direction [55]. The pre-treatment experiment showed that ALDH activity was increased in the rats treated with the low dose (1 mg/kg) of the extract, but when the extract dose was increased to the middle dose (10 mg/kg), ALDH activity was reduced. If it was consistent with the previous study, the rats receiving the high dose (100 mg/kg) of the extract should have even lower ALDH activity. However, the present study found that the activity of ALDH was increased in the rats treated with the high dose (100 mg/kg) of the extract, but it was not statistically significantly different when compared to the control group. This result was possible because the middle dose (10 mg/kg) of the extract might be the maximum dose that can affect ALDH activity, or variations in the experimental animals in the group receiving the high dose (100 mg/kg) of the extract, resulting in increased ALDH activity. In the post-treatment experiment, there was a more pronounced increase in ALDH activity, several times greater than in the pre-treatment experiment, where all groups of rats received the extract. The increase in ALDH activity was possibly due to the stimulatory effect of ethanol rather than the extract; as mentioned above, the extract could reduce ADH activity, resulting in slowing down the metabolism of ethanol. Thus, the remaining ethanol in the liver might stimulate ALDH activity. Furthermore, there was a trend towards a decrease in blood acetate levels in both experiments, with the greatest decrease occurring in the pre-treatment experiment at the third hour, indicating that administration of the leaf extract of *T. laurifolia* prior to ethanol exposure could reduce blood acetate levels, which was expected to result in reducing hangover symptoms, as previous studies have demonstrated that acetate is associated with hangover headaches [12].

The liver, the largest internal organ in the body, plays an important role in maintaining homeostasis of the body and removing toxins from the bloodstream [56]. Damage to the liver results in increased levels of liver enzymes released into the bloodstream [57]. Therefore, measuring liver enzyme levels, especially AST, ALT, and ALP, can indicate possible liver-related health problems [58]. Excessive alcohol consumption is one cause of liver cell damage [59,60]. Elevated levels of liver enzymes have been reported in people who consume alcoholic drinks and animal models of ethanol-induced hepatotoxicity [15,61]. In addition, a previous study has similarly reported that Wister albino rats force-fed with 40% ethanol continuously for 21 days developed hepatotoxicity with significantly increased liver enzyme activities of AST, ALT, as well as ALP [62]. In the pre-treatment experiment of the present study, administration of the leaf extract of *T. laurifolia* prior to ethanol exposure demonstrated hepatoprotective effects by preventing ethanol-induced changes in liver enzyme levels. In the post-treatment experiment, the administration of the leaf extract of *T. laurifolia* to the rats after receiving ethanol ameliorated the ethanol-induced liver damage by returning elevated levels of liver enzymes, especially AST and ALT, to normal levels. Similarly, the ability of the aqueous extract of *T. laurifolia* leaves to normalize liver enzyme levels has been previously reported when the extract was administered to Syrian hamsters after infection with the human liver fluke (*Opisthorchis viverrini*) [63]. Additionally, previous studies have shown the hepatoprotective effects of caffeic acid and rosmarinic acid by ameliorating the elevated liver enzyme levels when co-administration with ethanol in rats [64,65]. Vicenin-2 has also been found to ameliorate LPS-induced liver damage in mice [66].

An imbalance of free radicals and antioxidants in the body causes oxidative stress, leading to cellular inflammation, cell damage, and degeneration, which increases the risk of various health problems and diseases [67]. Alcohol consumption can stimulate the production of free radicals in the body and cause oxidative stress, which has adverse effects on various organs in the body, including the liver [68]. Alcohol-induced oxidative stress causes liver inflammation, as well as leads to hepatic fat accumulation and lipid peroxidation, which eventually results in liver damage [16]. Both endogenous and exogenous antioxidants can play a crucial role in maintaining the body’s redox balance and counteracting oxidative stress. Endogenous antioxidants include enzymatic antioxidants such as SOD and CAT and non-enzymatic antioxidants such as glutathione [69]. Different SOD isoforms can be found throughout the body, but the most abundant SOD isoforms in the liver are copper-zinc SOD (Cu/Zn-SOD) and manganese SOD (Mn-SOD) [70,71]. SOD has the function of converting the highly destructive free radical superoxide to the less destructive hydrogen peroxide, which is then converted into water and oxygen by CAT. Glutathione is a non-enzymatic antioxidant produced in the liver that can counteract free radicals and also acts as a cofactor for enzymatic antioxidants [72]. In the pre-treatment experiment, all groups of rats receiving the leaf extract of *T. laurifolia* prior to ethanol exposure had decreased SOD activity, whereas CAT activity was reduced, but no statistically significant difference was seen when compared to the control group. These results indicated that pre-treatment with all doses of the extract could protect against ethanol-induced oxidative stress, which was possible because phenolic and flavonoid compounds in the extract, such as caffeic acid, rosmarinic acid, and vicenin-2, have been demonstrated to possess antioxidant properties by acting as free radical scavengers, resulting in decreased endogenous SOD and CAT activities [20,73]. In the post-treatment experiment, administration of middle and high doses of the leaf extract of *T. laurifolia* to the rats increased SOD activity, while only the high dose of the extract increased CAT activity, thus indicating that if the rats received ethanol first, the subsequently administered extract had to be at the high dose to be able to both increase SOD and CAT activities. These results suggested that in the presence of oxidative stress caused by ethanol administration, endogenous SOD and CAT, as well as the subsequently administered extract, might stimulate the synthesis and/or activity of SOD and CAT [20,74]. In the pre-treatment experiment, total glutathione levels were increased in the rats given the low- and middle-dose extracts. This result might be due to the low content of the active compounds that exhibit antioxidant properties in the leaf extract of *T. laurifolia*; thus, the oxidative stress induced by ethanol exposure stimulated an increase in the synthesis of endogenous glutathione, resulting in increased total glutathione levels. On the other hand, total glutathione levels in the rats receiving the high dose of the extract were not different from the control group, probably due to the high content of the active compounds in the extract, which have antioxidant activities that scavenge free radicals. Therefore, the synthesis of endogenous glutathione was reduced and/or inactive, resulting in no significant increase in total glutathione levels. In the post-treatment experiment, the total glutathione levels were not significantly different either when comparing the groups receiving all doses of the extract with the control group or when comparing the groups receiving different doses of the extract. These effects might be due to the oxidative stress induced by the initial high ethanol exposure (5 g/kg body weight), stimulating endogenous glutathione synthesis, and subsequent administration of the extract, which might not have had any effect on this process, resulting in no difference in the total glutathione levels in all groups of rats. Previously, a return to baseline levels of hepatic glutathione by 24 h has been reported in mature mice after a single administration of 5 g/kg ethanol [75].

The present study was conducted on 8-week-old experimental rats. Further studies in various age groups of experimental animals were required before clinical testing of the efficacy and utility of the leaf extract of *T. laurifolia* in humans. In addition, the long-term safety of this extract, such as sub-chronic toxicity and chronic toxicity studies, must be evaluated.

## 4. Materials and Methods

### 4.1. Plant Material and Extract Preparation

The fresh leaves of *T. laurifolia* were collected from Sa Kaeo province, Thailand. The botanical and taxonomical characteristics of the plant specimen were identified according to the Thai Herbal Pharmacopoeia (THP) 2017 [76]. The plant specimen was identified by Dr. Piyanuch Rojsanga. A voucher specimen (RJ160401) was deposited at the Department of Pharmaceutical Chemistry, Faculty of Pharmacy, Mahidol University, Bangkok, Thailand. The fresh leaves were washed and dried in a hot-air oven at 60 °C for 8 h. After that, the dried leaves were ground into powder using an electronic mill (20 mesh sieve). The plant material was prepared and identified by TLC, the method outlined in the Thai Herbal Pharmacopeia [76], for quality control.

Qualified plant material was extracted by boiling the dried leaves in water (1:10, *w*/*v*) at 100 °C for 2 h, repeated twice. The filtrate was collected and then spray-dried (yield: 18.2% *w*/*w*). Then, the leaf extract was examined by using HPLC. The quality control of the leaf extract was evaluated in terms of appearance, weight loss on drying, and marker contents.

### 4.2. Chemical Composition Analysis by TLC

The leaf powder was boiled in water for 30 min and evaporated in a water bath until completely dry. The dried leaves of *T. laurifolia* were reconstituted by adding methanol (Merk, Darmstadt, Germany): water (50:50, *v*/*v*), and then 8 µL of this solution was spotted onto the TLC plate. Next, the TLC plate was developed with the mobile phase chloroform/methanol/formic acid (70:30:5, *v*/*v*) [76]. After that, the TLC plate (Merck, Darmstadt, Germany) was dried for 30 min in the fume hood and then analyzed using NP/PEG reagent (Sigma-Aldrich, St. Louis, MO, USA). The chromatogram was detected under a UV light detector (Camag, Muttenz, Switzerland) at 366 nm.

### 4.3. Quantitative Analysis of Marker Contents by HPLC

The leaf extract of *T. laurifolia* was dissolved in distilled water at a concentration of 2 mg/mL, with sonication for 10 min, and then the solutions were filtered using a 0.45 μm PTFE syringe filter (CNW Technologies, Shanghai, China). The chromatographic condition was adapted from Ruangpayungsak et al. [77]. The components of the HPLC system included a CBM-20A controller (Shimadzu, Kyoto, Japan), LC-20AD quaternary pump (Shimadzu, Kyoto, Japan), DGU-20A 5R degasser (Shimadzu, Kyoto, Japan), SIL-20AC HT autosampler (Shimadzu, Kyoto, Japan), CTO-20AC column oven (Shimadzu, Kyoto, Japan), and SPDM20A diode array detector (Shimadzu, Kyoto, Japan). Caffeic acid and rosmarinic acid, as marker contents, were quantitatively determined using X-terra C18, 150 × 3.9 mm i.d., 5 µm (Water, Milford, MA, USA). The gradient elution mode of 0.02% *ortho*-phosphoric acid (Merck, Darmstadt, Germany) in water (A) and acetonitrile (Merk, Darmstadt, Germany) (B) was used as the mobile phase [77]. A gradient program was used as follows: 10% B (0 min), 15% B (3 min), 20% B (15 min), 25% B (19–21 min), 30% B (25 min), 70% B (30 min), and 10% B (35–40 min). The analysis was performed at a flow rate of 1 mL/min at room temperature using a detection wavelength of 330 nm. A 20 μL sample was injected into the HPLC system. The ranges of analyses were 1.0–41.3 μg/mL for caffeic acid and 0.9–36.8 μg/mL for rosmarinic acid. Moreover, the limit of detection (LOD) and limit of quantitation (LOQ) were reported as 0.25 and 0.83 μg/mL for caffeic acid, as well as 0.26 and 0.86 μg/mL for rosmarinic acid, respectively.

### 4.4. Experimental Animal Housing and Care

The experimental animals were male Sprague–Dawley rats aged 8 weeks weighing 350 ± 20 g, which were purchased from the National Laboratory Animal Center, Mahidol University, Nakhon Pathom, Thailand. The rats were housed in a room with a temperature of 24 ± 1 °C and a 12 h light/dark cycle. All rats had free access to drinking water and a standard rodent-pelleted diet. They were acclimated for at least 7 days before the experiments began. All procedures used in this study were approved by the Animal Ethics Committee of the Faculty of Medicine, Chiang Mai University, Thailand, on 2 April 2015, with approval number 07/2558.

### 4.5. Experimental Procedure

This study was conducted using forty-eight male rats, which were fasted for 12 h and deprived of water for an additional hour before the experiment. The animals were randomly assigned to two experimental protocols to evaluate the effects of the *T. laurifolia* leaf extract when administered either before (pre-treatment) or after (post-treatment) alcohol exposure. Each experiment consisted of four groups, with six rats per group.

In the pre-treatment experiment (*n =* 24), the rats were first administered *T. laurifolia* leaf extract via oral gavage at doses of 1, 10, or 100 mg/kg body weight or distilled water as the vehicle control. Thirty minutes later, all animals received 40% ethanol (Merk, Darmstadt, Germany) at a dose of 5 g/kg body weight by oral gavage. The groups were assigned as follows:

Group 1A: distilled water + 40% ethanol (control group).

Group 2A: *T. laurifolia* leaf extract 1 mg/kg + 40% ethanol.

Group 3A: *T. laurifolia* leaf extract 10 mg/kg + 40% ethanol.

Group 4A: *T. laurifolia* leaf extract 100 mg/kg + 40% ethanol.

In the post-treatment experiment (*n =* 24), a separate set of rats was first administered 40% ethanol (5 g/kg body weight) by oral gavage. Thirty minutes after ethanol ingestion, they received either the *T. laurifolia* leaf extract at doses of 1, 10, or 100 mg/kg body weight or distilled water as a control. The groups were assigned as follows:

Group 1B: 40% ethanol + distilled water (control group).

Group 2B: 40% ethanol + *T. laurifolia* leaf extract 1 mg/kg.

Group 3B: 40% ethanol + *T. laurifolia* leaf extract 10 mg/kg.

Group 4B: 40% ethanol + *T. laurifolia* leaf extract 100 mg/kg.

### 4.6. Evaluation of Locomotor Activity

Locomotor activity assessments were performed by placing the rats in a square plexiglass chamber (17.5 × 18 × 12 inches) with a transmitted infrared light beam and connected to a data recorder. All rats were assessed for locomotor activity 30 min before administration of the test substances. In the pre-treatment experiment, the measurement of locomotor activity was conducted 30 min after the rats received the leaf extract of *T. laurifolia* and then 30 min after feeding the rats with ethanol. In the post-treatment experiment, locomotor activity was measured 30 min after the rats were administered ethanol. Next, locomotor activity measurements were performed again 30 min after the leaf extract of *T. laurifolia* was given to the rats.

### 4.7. Measurement of Ethanol and Acetate Levels

In both experiments, blood samples were collected from lateral tail veins at 0.5, 1, 3, and 5 h [78]. Ethanol and acetate levels in the blood samples were analyzed using an ethanol assay kit, Bioassay™, high sensitivity (United States Biological, Swampscott, MA, USA; Cat. No. E8476-55), and an acetate colorimetric assay kit (Sigma-Aldrich, St. Louis, MO, USA; Cat. No. MAK086), respectively.

### 4.8. Measurement of Alcohol-Metabolizing Enzymes

Twenty-four hours after administering the ethanol or leaf extract of *T. laurifolia* in the pre-treatment or post-treatment experiments, respectively, the rats were sacrificed with thiopental sodium (150 mg/kg, intraperitoneal injection). The death of the rats was confirmed by assessing the pulse, breathing, corneal reflex, toe pinch, and mucous membranes [79]. Liver tissues were collected and then homogenized for detection of the activities of ADH and ALDH using the NAD-ADH reagent multiple test vial (Sigma-Aldrich, St. Louis, MO, USA; Cat. No. N7160) and the aldehyde dehydrogenase activity colorimetric assay kit (Sigma-Aldrich, St. Louis, MO, USA; Cat. No. MAK082), respectively.

### 4.9. Measurement of Liver Enzyme Levels

The rats were euthanized by intraperitoneal injection of 150 mg/kg thiopental sodium 24 h after receiving ethanol in the pre-treatment experiment or after being given the leaf extract of *T. laurifolia* in the post-treatment experiment. The pulse, breathing, corneal reflex, toe pinch, and mucous membranes were verified for confirmation of death [79]. After that, blood samples were collected from the hearts by cardiac puncture into serum clot activator tubes (Hebei Xinle Sci&Tech Co., Ltd., Shijiazhuang, Hebei, China) [78]. Then, it was centrifuged at 3500 rpm for 10 min to obtain serum for the measurement of AST, ALT, and ALP levels by using an automated BX-3010 analyzer (Sysmex, Kobe, Japan).

### 4.10. Measurement of Antioxidant Enzyme Activities and Antioxidant Levels

Twenty-four hours after the rats were given ethanol in the pre-treatment experiment or the leaf extract of *T. laurifolia* in the post-treatment experiment, the rats were sacrificed by intraperitoneal injection of 150 mg/kg thiopental sodium, then the pulse, breathing, corneal reflex, toe pinch, and mucous membranes were checked to confirm the animal’s death [79]. Next, liver tissues were collected, and the activities of antioxidant enzymes consisting of SOD and CAT, as well as the level of total glutathione as an antioxidant in the liver homogenates, were measured using the OxiSelect™ superoxide dismutase activity assay (Cell Biolabs, Inc., San Diego, CA, USA; Cat. No. STA-340), the OxiSelect™ catalase activity assay kit (Cell Biolabs, Inc., San Diego, CA, USA; Cat. No. STA-341), and the OxiSelect™ total glutathione (GSSG/GSH) assay kit (Cell Biolabs, Inc., San Diego, CA, USA; Cat. No. STA-312), respectively.

### 4.11. Statistical Analysis

Statistical analysis was performed using SPSS Statistics Software 25 (SPSS Inc., Chicago, IL, USA). The data are expressed as the mean ± standard error of the mean (S.E.M). The normality test for statistical data used the Shapiro–Wilk test. In normally distributed data, a one-way analysis of variance (ANOVA) followed by Tukey’s multiple comparison tests was used to determine the statistical difference among groups. The data that were not normally distributed were analyzed using a Kruskal–Wallis nonparametric ANOVA test followed by a Dunn’s test. The statistical significance level was set at *p* < 0.05.

## 5. Conclusions

This study demonstrated that *T. laurifolia* alleviates hangover symptoms and exhibits hepatoprotective effects when administered to rats prior to ethanol exposure. Nonetheless, further studies are needed, particularly with an increased duration of ethanol and extract exposure, repeat-dose toxicity studies, and the development of the standardized extract into a standardized anti-hangover product before human clinical trials can proceed.

## Figures and Tables

**Table 1:** Quality control data of the dried leaves and the leaf extract of *T. laurifolia*.

Tests	Results
**The dried leaves of *T. laurifolia***	
Identification (TLC)	The sample solution exhibited bands at the same hRf values as caffeic acid and rosmarinic acid at hRf values of 85 and 71, respectively.
Caffeic acid (HPLC)	0.12 g/100 g dried leaves.
Rosmarinic acid (HPLC)	0.30 g/100 g dried leaves.
**The leaf extract of *T. laurifolia***	
Appearance	Dark brown in color with a distinct odor.
Weight loss on drying	5.98%.
Caffeic acid (HPLC)	0.14 g/100 g dried leaf extract.
Rosmarinic acid (HPLC)	0.24 g/100 g dried leaf extract.

**Table 2:** Blood ethanol levels of rats administered the leaf extract of *T. laurifolia* before ethanol administration.

Group	Blood Ethanol Levels (mmol/L)			
	0.5 h	1 h	3 h	5 h
Control (1A)	0.64 ± 0.30	3.86 ± 0.05	3.97 ± 0.11	1.61 ± 0.21
*T. laurifolia* extract 1 mg/kg (2A)	0.51 ± 0.02	3.27 ± 0.03	3.43 ± 0.03	1.61 ± 0.10
*T. laurifolia* extract 10 mg/kg (3A)	0.55 ± 0.02	3.06 ± 0.19	3.43 ± 0.37	1.61 ± 0.22
*T. laurifolia* extract 100 mg/kg (4A)	0.55 ± 0.03	2.84 ± 0.36 *	3.49 ± 0.12	1.61 ± 0.19

The data are expressed as mean ± S.E.M. (*n* = 6 rats per group). * *p* < 0.05 represents statistically significant differences from the control group.

**Table 3:** Blood ethanol levels of rats administered the leaf extract of *T. laurifolia* after ethanol administration.

Group	Blood Ethanol Levels (mmol/L)			
	0.5 h	1 h	3 h	5 h
Control (1B)	0.86 ± 0.09	2.25 ± 0.06	2.79 ± 0.19	2.04 ± 0.28
*T. laurifolia* extract 1 mg/kg (2B)	0.70 ± 0.14	2.25 ± 0.14	2.41 ± 0.04	1.34 ± 0.20
*T. laurifolia* extract 10 mg/kg (3B)	0.91 ± 0.11	1.82 ± 0.05 *	1.99 ± 0.07 *	0.86 ± 0.04 *
*T. laurifolia* extract 100 mg/kg (4B)	1.07 ± 0.23	2.36 ± 0.08	2.41 ± 0.04	1.40 ± 0.02

The data are expressed as mean ± S.E.M. (*n* = 6 rats per group). * *p* < 0.05 represents statistically significant differences from the control group.

**Table 4:** Blood acetate levels of rats administered the leaf extract of *T. laurifolia* before ethanol administration.

Group	Blood Acetate Levels (ng/µL)			
	0.5 h	1 h	3 h	5 h
Control (1A)	14.99 ± 1.23	27.51 ± 0.15	38.61 ± 0.61	41.63 ± 5.59
*T. laurifolia* extract 1 mg/kg (2A)	17.97 ± 0.09	28.57 ± 1.11	33.77 ± 0.75 *	35.64 ± 1.07
*T. laurifolia* extract 10 mg/kg (3A)	14.34 ± 0.21	18.99 ± 0.28 *	27.37 ± 0.73 *	36.16 ± 1.06
*T. laurifolia* extract 100 mg/kg (4A)	16.13 ± 0.22	23.83 ± 0.05 *	24.08 ± 0.11 *	34.82 ± 0.85

The data are expressed as mean ± S.E.M. (*n* = 6 rats per group). * *p* < 0.05 represents statistically significant differences from the control group.

**Table 5:** Blood acetate levels of rats administered the leaf extract of *T. laurifolia* after ethanol administration.

Group	Blood Acetate Levels (ng/µL)			
	0.5 h	1 h	3 h	5 h
Control (1B)	19.51 ± 0.47	25.91 ± 0.42	30.88 ± 1.22	30.58 ± 0.80
*T. laurifolia* extract 1 mg/kg (2B)	18.28 ± 0.97	26.14 ± 0.63	26.60 ± 1.70	28.23 ± 1.60
*T. laurifolia* extract 10 mg/kg (3B)	18.88 ± 0.69	22.07 ± 0.78 *	23.94 ± 0.42 *	29.75 ± 0.33
*T. laurifolia* extract 100 mg/kg (4B)	19.30 ± 0.19	24.47 ± 0.77	26.54 ± 0.81	28.83 ± 1.22

The data are expressed as mean ± S.E.M. (*n* = 6 rats per group). * *p* < 0.05 represents statistically significant differences from the control group.

**Table 6:** Liver enzyme levels of rats administered the leaf extract of *T. laurifolia* before ethanol administration.

Group	Liver Enzyme Levels (U/L)		
	AST	ALT	ALP
Control (1A)	121.67 ± 4.61	38.50 ± 3.04	139.83 ± 5.10
*T. laurifolia* extract 1 mg/kg (2A)	120.33 ± 7.26	38.00 ± 2.42	140.50 ± 5.02
*T. laurifolia* extract 10 mg/kg (3A)	125.83 ± 6.41	42.17 ± 3.13	133.17 ± 3.03
*T. laurifolia* extract 100 mg/kg (4A)	109.33 ± 4.71	35.67 ± 1.93	135.17 ± 4.25

The data are expressed as mean ± S.E.M. (*n* = 6 rats per group).

**Table 7:** Liver enzyme levels of rats administered the leaf extract of *T. laurifolia* after ethanol administration.

Group	Liver Enzyme Levels (U/L)		
	AST	ALT	ALP
Control (1B)	146.83 ± 12.26	40.17 ± 1.42	126.67 ± 4.80
*T. laurifolia* extract 1 mg/kg (2B)	115.33 ± 3.70 *	36.67 ± 2.89	129.83 ± 4.08
*T. laurifolia* extract 10 mg/kg (3B)	111.67 ± 4.43 *	33.83 ± 1.85	118.83 ± 4.73
*T. laurifolia* extract 100 mg/kg (4B)	108.83 ± 6.93 *	32.83 ± 1.19 *	114.67 ± 5.02

The data are expressed as mean ± S.E.M. (*n* = 6 rats per group). * *p* < 0.05 represents statistically significant differences from the control group.

## Data Availability

The original contributions presented in this study are included in the article. Further inquiries can be directed to the corresponding author.
